# Miscarriage and the Microbiome: Host Genetics, Immunity, and the Reproductive Tract Ecosystem

**DOI:** 10.3390/genes17030259

**Published:** 2026-02-25

**Authors:** Nektaria Zagorianakou, Stylianos Makrydimas, Efthalia Moustakli, Ioannis Mitrogiannis, George Makrydimas

**Affiliations:** 1 Department of Nursing, School of Health Sciences, University of Ioannina, Scientific Laboratory for Innovative Technologies in Internal Medicine, Preventive Medicine and Overall Care, 45500 Ioannina, Greece; zagorianakou@uoi.gr; 2 Medical School, Aristotle University of Thessaloniki, 54124 Thessaloniki, Greece; smakrydimas@gmail.com; 3 Department of Nursing, School of Health Sciences, University of Ioannina, 4th Kilometer National Highway Str. Ioannina-Athens, 45500 Ioannina, Greece; ef.moustakli@uoi.gr; 4 Harris Birthright Research Centre for Fetal Medicine, King’s College London, 16–20 Windsor Walk, London SE5 8BB, UK; ioannismitrogiannisgr@gmail.com; 5 Department of Obstetrics & Gynecology, University Hospital of Ioannina, 45500 Ioannina, Greece

**Keywords:** maternal–fetal interface, microbial dysbiosis, immune–microbial interactions, decidualization, placentation, epigenetic regulation, gene regulation, microbial metabolites

## Abstract

**Background/Objectives:** Pregnancy loss is a common and multifactorial complication of human reproduction, traditionally attributed to fetal chromosomal abnormalities, maternal anatomical and endocrine disorders, and immune dysfunction. Growing evidence now indicates that the maternal microbiome, particularly within the reproductive tract, plays a critical role in implantation, placental development, and the maintenance of immune tolerance during early pregnancy. Importantly, the influence of the microbiome on miscarriage appears to be strongly modulated by host genetic background and immune regulation. **Methods:** This narrative review summarizes current evidence linking alterations in the vaginal, endometrial, placental, and gut microbiomes to miscarriage, with a specific focus on host genetics and immune–microbial interactions. **Results:** We discuss how genetic variation in innate and adaptive immune pathways, inflammatory signaling, and mucosal barrier function may shape host responses to microbial communities, thereby influencing susceptibility to PL. In addition, we highlight emerging data on microbiome-driven regulation of gene expression and epigenetic modifications in the endometrium and decidua, emphasizing the role of microbial metabolites in immune tolerance and placental function. **Conclusions:** By integrating findings from microbiome research, host genomics, immunology, and epigenetics, this review proposes a framework in which miscarriage is viewed as a consequence of disrupted host–microbe crosstalk rather than isolated pathology. Finally, we address key methodological challenges and outline future research directions aimed at advancing mechanistic understanding and translational applications.

## 1. Introduction

Pregnancy loss represents a heterogeneous group of conditions and constitutes a significant reproductive health burden, affecting up to 15–20% of clinically recognized pregnancies [1]. Pregnancy loss is clinically divided into two categories: recurrent pregnancy loss (RPL) and spontaneous pregnancy loss (SPL). These two conditions differ significantly in terms of frequency, etiology, and underlying molecular mechanisms [2,3].

Most cases of sporadic pregnancy loss are caused by fetal chromosomal abnormalities resulting from de novo meiotic or mitotic mistakes, especially in the early stages of gestation [4,5,6]. In contrast, RPL, defined as the loss of two or more pregnancies, shows a lower prevalence of embryonic aneuploidy and is increasingly associated with maternal factors, including immune dysregulation, impaired endometrial receptivity, and alterations of the reproductive tract microenvironment [2,7].

Despite standardized diagnostic evaluations, up to 40-50% of RPL cases remain unexplained, highlighting the limitations of current etiological frameworks that rely solely on genetic, anatomical, endocrine, or thrombophilic factors [2]. The distinction between spontaneous and RPL is crucial from a clinical and biological standpoint since the mechanisms linked to RPL involve potentially modifiable maternal pathways and go beyond random genetic occurrences [8].

Proper maternal–fetal immunological tolerance is crucial for successful implantation and early placental development, especially at the maternal–fetal interface, where disruptions in immune cells and inflammation can lead to pregnancy loss [9,10,11]. As a result, there is mounting evidence that immunological dysregulation and persistent inflammatory activation play a major part in the etiology of unexplained RPL [12].

Simultaneously, the human microbiome has become a crucial modulator of inflammatory tone and immunological homeostasis, with growing significance for successful reproduction [13,14]. Local inflammation, decreased endometrial receptivity, and unfavorable reproductive outcomes, such as RPL, have been linked to vaginal and endometrial dysbiosis, which is frequently characterized by decreased Lactobacillus dominance and increased microbial diversity [15,16].

Emerging evidence challenges the long-held belief of uterine sterility, suggesting that a low-biomass but physiologically active endometrial microbiome may influence immunological pathways essential for implantation and early pregnancy [17,18]. The idea that a systemic microbiome–immune axis influences maternal–fetal interactions is supported by the fact that changes in extra-genital microbiome habitats, such as the gut and oral microbiomes, have also been connected to pregnancy loss outside of the reproductive tract [19].

Collectively, these findings identify immune–microbiome interactions as a critical and underexplored component of RPL pathophysiology, distinct from the mechanisms underlying spontaneous miscarriage. However, the existing literature remains fragmented across immunology, reproductive biology, and microbiome research, limiting the translation of emerging insights into clinical practice [20,21].

The aim of this narrative review is to synthesize current evidence on the interplay between immune dysregulation and microbiome alterations in recurrent pregnancy loss, with a specific focus on vaginal, endometrial, and systemic microbial ecosystems and their molecular crosstalk with maternal immune pathways. We further seek to contrast these mechanisms with those underlying SPL, highlight gaps in existing knowledge, and discuss the implications for biomarker discovery and targeted therapeutic strategies in women with unexplained RPL. Although diagnostic definitions of RPL vary among professional societies and geographic regions, the biological pathways discussed here are broadly applicable across these classifications.

## 2. Literature Search Strategy

This narrative review synthesizes current evidence on the interplay between host genetics, immune dysregulation, and microbiome alterations in recurrent pregnancy loss (RPL). The relevant literature was identified through searches of PubMed, Scopus, and Web of Science using combinations of keywords including “recurrent pregnancy loss,” “miscarriage,” “microbiome,” “vaginal microbiota,” “endometrial microbiome,” “immune tolerance,” “decidualization,” “host genetics,” “cytokines,” “natural killer cells,” “epigenetics,” and “maternal–fetal interface.”

Priority was given to peer-reviewed original research articles and review papers published primarily between 2000 and 2025, with inclusion of seminal earlier studies where necessary to provide mechanistic context. Studies were selected based on their relevance to microbiome composition, immune regulation, genetic susceptibility, or epigenetic mechanisms implicated in sporadic or recurrent pregnancy loss.

Additional relevant publications were identified through manual screening of reference lists. The retrieved literature was synthesized thematically according to biological mechanisms and anatomical compartments to support the integrated framework proposed in this review. No new experimental data were generated or analyzed.

## 3. RPL as a Distinct Pathophysiological Entity Beyond Embryonic Aneuploidy

RPL is a unique biological phenomenon that is very different from an occasional miscarriage. While solitary pregnancy loss is mostly caused by embryonic aneuploidy, recurrent instances increasingly involve persistent maternal variables, requiring integrative models that go beyond fetal chromosomal abnormalities. Chromosomal and genetic factors are essential for understanding the role of classical genetics in RPL, as well as its limitations [2,22].

### 3.1. Genetic and Chromosomal Contributions to Pregnancy Loss

The most common cause of sporadic pregnancy loss, especially in the first trimester, is chromosomal abnormalities; up to 50–70% of early miscarriages include embryonic aneuploidy. These anomalies are strongly associated with advanced maternal age and are usually caused by de novo meiotic or mitotic errors. As a result, sporadic pregnancy loss is often seen as a random occurrence that is mostly unrelated to biological malfunction in the mother [23,24].

However, the role of embryonic aneuploidy in repeated miscarriages seems to be much smaller. Several studies have demonstrated that a significant proportion of miscarriages in women with RPL are chromosomally normal, suggesting alternative pathogenic mechanisms beyond fetal genetic abnormalities. Data from cytogenetic tests and preimplantation genetic testing, which show comparable aneuploidy frequencies in embryos from women with and without RPL, further corroborate this finding [24,25,26].

### 3.2. Predominance of Maternal Factors in RPL

The focus of study has switched to maternal factors that contribute to pregnancy failure due to the diminished explanatory value of embryonic chromosomal abnormalities in RPL [27].

Immune dysregulation, reduced endometrial receptivity, poor decidualization, and ongoing inflammation at the maternal–fetal interface are a few examples. Since the semi-allogeneic embryo must avoid immune-mediated rejection, maternal immunological tolerance is crucial for effective implantation and early placental development [11,12].

Changes in immune cell populations, cytokine profiles, and inflammatory pathways are commonly seen in women with RPL, suggesting that immune imbalance plays a key role in the disease’s development [28,29]. Crucially, the recurring nature of pregnancy loss seen in RPL may have a biological basis due to the potential persistence of these maternal anomalies across pregnancies. These contrasts sharply with sporadic pregnancy loss, where the recurrence risk remains low and largely driven by chance rather than sustained maternal dysfunction [2].

### 3.3. Implications for Mechanistic and Translational Research

Recognizing RPL as a distinct condition has important implications for clinical management and research design. Mechanistic studies focused solely on fetal genetics are unlikely to fully explain the etiology of RPL, especially in unexplained cases [2,30]. Instead, increasing evidence suggests that early pregnancy maintenance and implantation are affected by interactions between the uterine environment and maternal immune dysregulation. In this context, the reproductive tract microbiome is emerging as a key factor in immune activation and endometrial function, offering new insights into the causes of recurrent miscarriages [31,32].

## 4. Genetic Architecture of Pregnancy Loss and Its Limitations in RPL

The most well-researched causes of pregnancy loss are genetic variables, which have historically served as the main explanation, especially in cases of sporadic miscarriage. However, a strictly gene-centric model has significant drawbacks because the genomic architecture causing pregnancy loss varies significantly between sporadic and recurrent cases. This section explains why most cases of RPL cannot be explained by traditional genetic pathways and summarizes the proven role of embryonic and parental genetic disorders in pregnancy loss [6].

### 4.1. Embryonic Chromosomal Abnormalities in SPL

Aneuploidy is found in about 50–70% of early miscarriages, and embryonic chromosomal abnormalities are the most common cause of sporadic pregnancy loss, especially during the first trimester. The majority of these defects are caused by de novo meiotic nondisjunction events and mitotic mistakes, which are more common as mothers age. Therefore, it is generally accepted that fetal genetic abnormalities, rather than persistent maternal illness, are the primary cause of random pregnancy loss, which is a stochastic event [24]. The majority of chromosomal abnormalities seen in spontaneous miscarriages are caused by autosomal trisomies, monosomy X, and polyploidy, according to extensive cytogenetic and sequencing-based research [33].

Preimplantation genetic testing for aneuploidy (PGT-A) data, which shows significant rates of chromosomal imbalance among embryos that fail to implant or cause early pregnancy loss, has further corroborated these conclusions [34,35].

### 4.2. Parental Genetic Factors in RPL

RPL has a different genetic profile than random pregnancy loss, and the recurrence of the condition cannot be entirely explained by embryonic aneuploidy alone. About 2–5% of couples with RPL have balanced chromosomal rearrangements, such as reciprocal and Robertsonian translocations, which are among the most well-established genetic risk factors. In some RPL cases, other parental genetic factors, such as copy number variants (CNVs) and rare monogenic disorders, have been suggested; however, their overall impact remains limited and not well understood [5,6].

Crucially, a significant percentage of couples with RPL do not have a causal genetic defect found by thorough genetic evaluation, including karyotyping and, when appropriate, molecular testing [5].

### 4.3. Euploid Pregnancy Loss and the Limits of a Gene-Centric Model

The high frequency of chromosomally normal (euploid) losses in affected women is a crucial finding that contradicts a purely gene-centric hypothesis of RPL [36]. Multiple studies using next-generation sequencing, comparative genomic hybridization, and conventional karyotyping have shown that a substantial proportion of pregnancy losses in RPL occur without detectable chromosomal abnormalities [37].

Additionally, aneuploidy rates in embryos from women with RPL undergoing PGT-A and assisted reproduction are frequently similar to those seen in fertile controls, indicating that intrinsic embryonic genetic abnormalities are not the only cause of recurrent loss [38,39]. Collectively, these findings indicate that although genetic variables are important for sporadic pregnancy loss and contribute to a portion of RPL instances, they are not enough to account for most unexplained RPL [40].

### 4.4. Toward an Integrated Genetic and Environmental Framework

The necessity to take into account other biological layers that influence gene expression and pregnancy outcome is shown by the limited ability of classical genetic variables to explain unexplained RPL. There is increasing recognition that environmental factors, such as the reproductive tract microbiota, maternal immune tolerance, and epigenetic regulation, act as important modifiers of reproductive outcomes, either independently or in combination with genetic variation [41,42,43].

**Figure 1:**
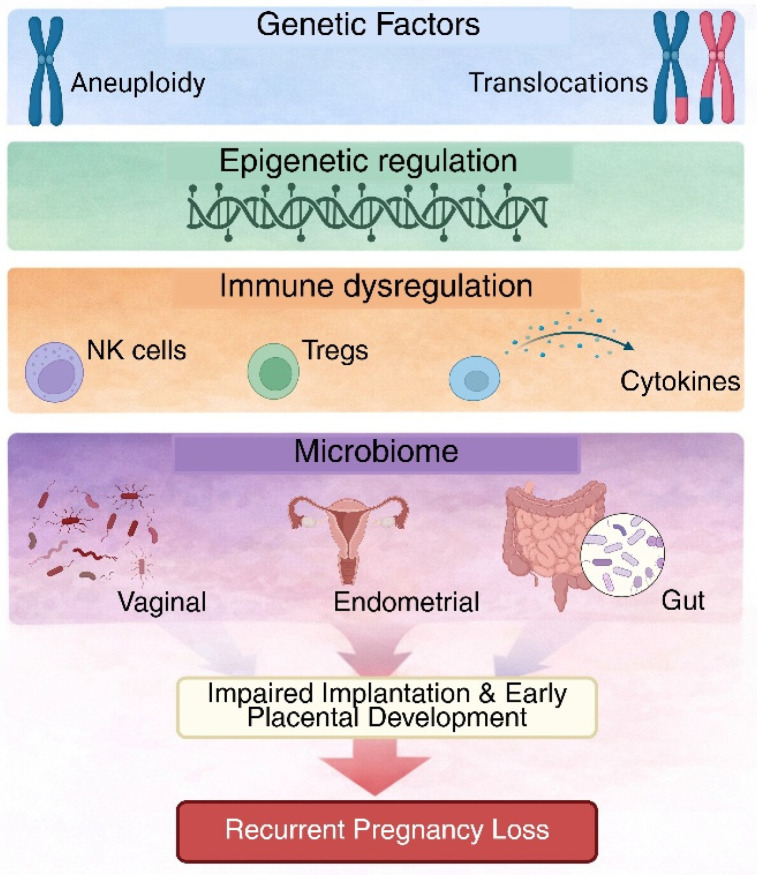
Multilayered pathophysiology of RPL. This diagram shows the various factors involved in RPL. While genetic issues like embryonic aneuploidy contribute to some cases, they do not explain most cases of unexplained RPL. Other factors, such as immune dysregulation, epigenetic changes, and the microbiome in the reproductive and extra-reproductive areas (like the gut and oral cavity), influence inflammation, endometrial receptivity, and early placental development, leading to pregnancy loss.

Investigating immune–microbiome crosstalk in RPL is therefore justified by modern models of RPL, which provide an integrated framework in which genetic susceptibility interacts with immunological and microenvironmental variables to determine pregnancy outcome [41,44]. When combined, these findings show that although genetic variables are important in cases of random pregnancy loss and contribute to some cases of RPL, they are not enough to account for most unexplained RPL [5]. Table 1 presents a comparison of known genetic factors in spontaneous vs. RPL, and Figure 1 provides a schematic summary of the wider interaction between genetic, immunological, and microbiome-related factors.

## 5. Maternal–Fetal Immune Tolerance and Immune Dysregulation in RPL

The development of precisely controlled maternal–fetal immunological tolerance, which enables the semi-allogeneic embryo to avoid immune-mediated rejection while preserving efficient host defense, is essential for successful implantation and early placental development. Decidual immune cells, cytokine networks, and endometrial stromal cells work together to coordinate this carefully regulated immunological balance at the maternal–fetal interface [48,49].

A pro-inflammatory milieu that jeopardizes implantation and early placental development is created when these immune regulatory mechanisms are disrupted in women who experience RPL, according to mounting data. Immune dysregulation interacts with genetic vulnerability, epigenetic control, and environmental factors to influence pregnancy outcome within an integrated pathophysiological framework [12].

As schematically illustrated in Figure 1, alterations in maternal immune tolerance represent a central node through which genetic and microbiome-related factors converge to influence inflammatory signaling, endometrial receptivity, and decidualization processes. Therefore, understanding the immunological mechanisms underlying RPL is essential for clarifying disease pathophysiology and identifying potential diagnostic and therapeutic targets.

### 5.1. Immune Landscape of the Maternal–Fetal Interface

Immune tolerance for the semi-allogeneic embryo must be developed in the maternal–fetal interface, a special immunological niche, while maintaining efficient host defense systems. Decidual natural killer (dNK) cells, macrophages, and T lymphocytes are among the immune cell types that make up this unique immunological milieu, which is found inside the decidua and is distinguished by a unique composition and functional polarization [50,51]. Approximately 50–70% of decidual leukocytes are decidual natural killer cells, which comprise the majority of immune cells during the early stages of pregnancy.

In contrast to peripheral cytotoxic NK cells, dNK cells exhibit reduced cytotoxic activity and secrete a broad range of cytokines, chemokines, and angiogenic factors that promote trophoblast invasion, spiral artery remodeling, and placental development [52,53,54].

Macrophages exhibit functional flexibility during the course of pregnancy and are the second most prevalent immune cell type at the maternal–fetal interface. Decidual macrophages are mostly polarized toward an anti-inflammatory and tissue-remodeling phenotype during the early stages of pregnancy, which helps with immunological tolerance, extracellular matrix remodeling, and apoptotic cell clearance [50,55,56].

T lymphocytes, particularly regulatory T cells (Tregs), proliferate during early pregnancy and suppress excessive inflammatory responses, making them essential for maintaining immunological homeostasis. Tregs protect the developing embryo from immune-mediated rejection by suppressing effector T cells and promoting maternal immune tolerance to fetal antigens [57].

The coordinated interactions of decidual NK cells, macrophages, and T lymphocytes create a tightly regulated immunological milieu that supports implantation and early placental development. The decidual milieu can become more pro-inflammatory when this balance is upset, which raises the chance of implantation failure and repeated pregnancy loss. This can occur through alterations in immune composition, phenotype, or functional activity [11,50].

### 5.2. Immune Abnormalities at the Maternal–Fetal Interface in RPL

A growing body of research suggests that repeated miscarriages are linked to a change toward a pro-inflammatory decidual milieu by upsetting the strictly controlled immunological balance at the maternal–fetal interface. Several cellular compartments and communication pathways that are essential for implantation and early placental development are impacted by these immunological changes [58,59].

Alterations in decidual and peripheral NK cells are among the most frequently reported immunological findings in RPL. When compared to women who had successful pregnancies, women with RPL often show enhanced NK cell cytotoxicity, altered NK cell receptor expression, and poor functional polarization. These alterations may impair spiral artery remodeling and trophoblast invasion, processes that depend on the regulatory, rather than cytotoxic, functions of decidual NK cells [54,60].

The pathophysiology of RPL has also been firmly linked to imbalances in T-cell subsets. In women with RPL, there is an increase in pro-inflammatory effector T-cell populations along with decreased frequencies and compromised suppressive activity of Tregs. This shift is often reflected in a higher Th1/Th17-to-Treg ratio and increased levels of inflammatory cytokines, including interleukin-17, interferon-γ, and tumor necrosis factor-α [61,62].

Concurrently, there seems to be a change in macrophage polarization at the maternal–fetal interface in RPL. Decidual macrophages in RPL have been shown to exhibit a stronger pro-inflammatory profile, which may enhance local inflammatory signaling and impede endometrial receptivity, as opposed to the mostly anti-inflammatory and tissue-remodeling phenotype seen in normal pregnancy [63,64].

**Figure 2:**
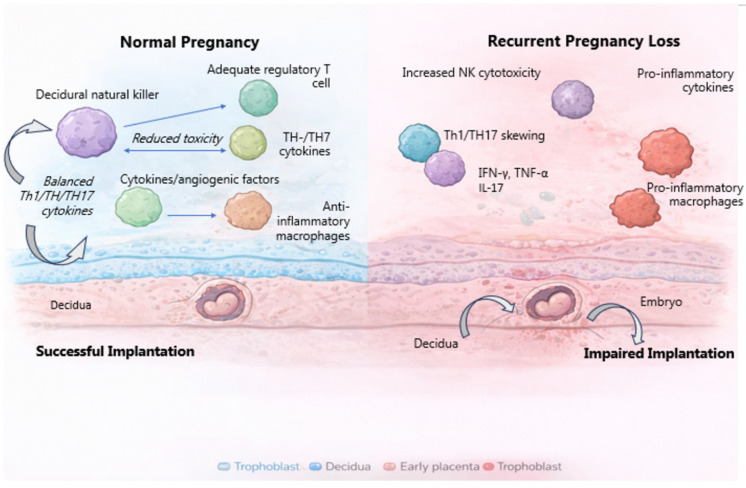
Schematic comparison of the immune landscape at the maternal–fetal interface during normal pregnancy and RPL. Normal pregnancy is characterized by regulatory decidual NK cell function, adequate regulatory T-cell abundance, balanced cytokine signaling, and anti-inflammatory macrophage polarization. In contrast, RPL is associated with increased NK cell cytotoxicity, reduced regulatory T-cell activity, Th1/Th17 skewing, and pro-inflammatory macrophage polarization, collectively contributing to impaired implantation and early placental development.

When these immunological anomalies converge, they create a hostile decidual environment characterized by high levels of inflammation, impaired immune tolerance, and inadequate communication between trophoblasts and maternal immune cells. Importantly, these immunological changes often persist across pregnancies, providing a biological explanation for the repeated pregnancy loss observed in RPL [65]. These contrasting immune landscapes at the maternal–fetal interface in normal pregnancy versus RPL are schematically summarized in Figure 2.

### 5.3. Immune–Microbiome Crosstalk in RPL

Microbial signals from the reproductive and extra-reproductive tract microbiomes constantly influence the maternal immune system, which does not function in a vacuum. Increasing evidence indicates that alterations in microbiome composition influence both systemic and local immune responses, thereby affecting endometrial receptivity and maternal–fetal immune tolerance. The vaginal microbiota plays a key role in maintaining immunological homeostasis within the female reproductive tract [66,67]. Vaginal dysbiosis, characterized by reduced Lactobacillus abundance and increased microbial diversity, has been associated with elevated pro-inflammatory cytokine production and adverse reproductive outcomes, including RPL. In contrast, a Lactobacillus-dominated vaginal environment is associated with low inflammatory tone, intact epithelial barrier function, and protection against pathogenic colonization [68,69].

Microbial products from dysbiotic vaginal communities can activate innate immune pathways through pattern recognition receptors, such as Toll-like receptors, leading to increased production of inflammatory mediators that may disrupt implantation and early placental development. The functional polarization of decidual immune cells, such as macrophages and natural killer cells, may be further impacted by these immunological disruptions, intensifying local inflammatory reactions [21,62].

There may be a low-biomass but physiologically significant endometrial microbiome outside of the vaginal compartment that can affect immunological signaling at the mother–fetus contact, according to new research. Alterations in the endometrial microbiome are associated with impaired decidualization, reduced implantation potential, and early pregnancy loss [70].

Immune dysregulation in RPL may be caused by extra-reproductive microbiomes, especially the gut microbiome, in addition to local impacts [41]. Inflammatory signals and metabolites produced from the gut microbiota can have systemic immunomodulatory effects that affect T-cell development, regulatory T-cell activity, and inflammatory cytokine production—all of which are essential for maintaining pregnancy [71,72].

Overall, these findings support the concept that microbiome dysbiosis alters immune regulatory processes at both the local and systemic levels, thereby contributing to RPL. Immune–microbiome crosstalk appears in this framework as a crucial mechanistic connection between environmental exposures and maternal immune dysregulation, offering a biologically tenable explanation for unexplained RPL as well as a possible target for upcoming diagnostic and treatment approaches [13,73].

## 6. Vaginal Microbiome Alterations in RPL

The vaginal microbiome is a key determinant of immune balance in the female reproductive tract and plays an essential role in reproductive health. Low vaginal pH, the integrity of the epithelial barrier, and the inhibition of pathogenic bacteria development are all linked to a Lactobacillus-dominated vaginal microbiome, which is thought to be the ideal state in women of reproductive age [74,75,76]. Healthy vaginal microbiomes are generally dominated by Lactobacillus species, especially Lactobacillus crispatus, Lactobacillus iners, Lactobacillus gasseri, and Lactobacillus jensenii, according to extensive sequencing-based research. These microbial communities are characterized by low inflammatory tone and minimal innate immune activation, creating conditions that support implantation and early pregnancy [77].

Ravel et al. showed that the vaginal microbiome of women of reproductive age may be categorized into several community state types based on the relative abundance of bacterial taxa, rather than fixed proportions of particular species, using 16S rRNA gene sequencing [78]. Reduced Lactobacillus abundance and increased microbial diversity characterize vaginal dysbiosis, which is associated with elevated vaginal pH and local inflammation. Negative reproductive consequences, such as implantation failure, premature birth, and pregnancy loss, have been repeatedly associated with such dysbiotic conditions [79].

There is growing evidence that women who experience repeated miscarriages are more likely to have vaginal dysbiosis than women who are still pregnant or have never experienced a miscarriage. In women with RPL, studies employing 16S rRNA gene sequencing have shown decreased Lactobacillus dominance and enrichment of anaerobic taxa frequently linked to bacterial vaginosis [80]. Vaginal dysbiosis has been linked to heightened inflammatory signaling, indicating a connection between altered microbial composition and immune dysregulation at the maternal–fetal interface. Such inflammatory signals may impair endometrial receptivity through microbial ascent or immune-mediated effects within the uterus [15,81].

The idea that a chronic microbial–immune imbalance contributes to disease recurrence is supported by accumulating data suggesting the association between vaginal microbiome makeup and pregnancy loss is more prominent in recurrent miscarriages than in random ones. All of these results point to vaginal microbiome dysbiosis as a crucial upstream factor that can influence immune responses and vulnerability to repeated miscarriages [41,80].

## 7. Endometrial Microbiome and Local Immune Modulation in RPL

The immunological and microbiological environment of the endometrium, the site of implantation and early placental development, is critical for successful pregnancy. Recent studies suggest that the endometrium harbors a low-biomass yet biologically active microbiota capable of shaping local immune responses [7]. Advances in high-throughput sequencing have enabled the detection of bacterial DNA in carefully collected endometrial samples, revealing microbial communities distinct from those of the lower reproductive tract. In reproductive health, the endometrial microbiome is typically characterized by low diversity and relative dominance of Lactobacillus species, a profile associated with immunological quiescence and successful implantation [7,67].

Conversely, implantation failure, early pregnancy loss, and RPL have been linked to endometrial dysbiosis, which is characterized by decreased Lactobacillus abundance and increased microbial diversity. Women with RPL have been reported to display endometrial microbial profiles that differ from fertile controls, indicating a potential role for local microbial imbalance [82,83]. Mechanistically, immune modulation at the mother–fetus interface can be directly influenced by the endometrial microbiome. Microbe-derived components can activate innate immune signaling through pattern recognition receptors expressed by endometrial epithelial and stromal cells, leading to altered cytokine production and immune recruitment. Decidualization, trophoblast–endometrium crosstalk, and implantation may all be hampered by such immune activation [67,84].

Immunological changes associated with endometrial dysbiosis may act independently or together with alterations in the vaginal microbiome. Although ascending microbial exposure is possible, evidence also suggests that local immune selection, rather than passive transfer from the lower genital tract, may shape endometrial microbial communities [85]. Interpretation of endometrial microbiome data requires careful attention to methodological limitations. The low microbial biomass of the endometrium increases susceptibility to contamination, underscoring the need for rigorous sampling procedures, appropriate controls, and cautious data interpretation. Despite these challenges, accumulating evidence supports a physiologically meaningful association between endometrial microbial composition, immune modulation, and reproductive outcomes [86,87].

All of these results suggest that endometrial microbiome dysbiosis may promote local immunological dysregulation at the implantation site, which could lead to RPL. The endometrium represents a key site where microbial and immune signals converge to influence early pregnancy success within the broader microbiome–immune axis [13].

## 8. Beyond the Reproductive Tract: Gut and Oral Microbiome Contributions to RPL

The maternal–fetal interface is directly impacted by the vaginal and endometrial microbiomes, but there is mounting evidence that extra-reproductive microbiomes also play a role in immune control during pregnancy [88,89]. Among these, the gut microbiota is a key modulator of inflammatory tone and systemic immunological homeostasis, which may have consequences for repeated miscarriages. The gut microbiota influences host immunity through multiple mechanisms, including modulation of innate immune signaling, regulation of T-cell differentiation, and production of immunologically active microbial metabolites [88,90].

Short-chain fatty acids and other microbe-derived products influence cytokine synthesis, inflammatory balance, and the growth of regulatory T cells. These activities are critical for preserving maternal–fetal immunological tolerance. Immune dysregulation and systemic inflammatory states have been linked to changes in gut microbiome composition, which may have an indirect impact on reproductive outcomes. Emerging data from pregnancy-related illnesses support a role for gut–immune interactions in modifying endometrial receptivity and placental development, despite the lack of direct evidence linking gut microbiome dysbiosis to RPL [13,20,91].

The oral microbiota has also been implicated in adverse pregnancy outcomes through systemic inflammatory pathways. Oral dysbiosis and periodontal disease are associated with increased circulating inflammatory mediators and bacterial translocation, which may influence immune responses at distant sites, including the uterus. Oral microbial dysbiosis may be a sign or modulator of systemic immunological imbalance, according to recent metagenomic analyses that have revealed unique oral microbiome patterns in women with a history of pregnancy loss [92,93].

These findings suggest that alterations in the oral microbiome may contribute to RPL through immune-mediated mechanisms, although direct causal relationships have not yet been established. When taken as a whole, these findings demonstrate the significance of systemic microbial impacts on maternal immune modulation and extend the microbiome–immune axis beyond the reproductive tract [94,95]. The gut and oral microbiomes may function as upstream modulators of immunological tolerance within an integrated framework, influencing vulnerability to RPL through interactions with the local reproductive tract microbiota [41].

## 9. Molecular and Inflammatory Mechanisms Linking Microbiome Dysbiosis to RPL

Microbiome dysbiosis can influence pregnancy outcomes through biochemical and inflammatory processes that directly affect immune tolerance, endometrial function, and early placental development. These processes function at the intersection of oxidative stress (OS), cytokine modulation, innate immunological signaling, and transcriptional regulation of genes essential for decidualization and implantation [96,97].

Microbe-derived components, such as lipopolysaccharide and other pathogen-associated molecular patterns, activate innate immune pathways through pattern recognition receptors, including Toll-like receptors, expressed by endometrial epithelial and immune cells [98,99]. When these pathways are activated, pro-inflammatory cytokines and chemokines are expressed more frequently as a result of downstream signaling cascades involving nuclear factor-κB (NF-κB) and associated transcription factors. It has been demonstrated that persistent activation of these inflammatory pathways hinders trophoblast invasion and interferes with communication between the embryo and the endometrium [62,100].

OS in the reproductive tract is closely linked to microbiome-induced inflammation. Excessive production of reactive oxygen species can damage cellular macromolecules, disrupt mitochondrial function, and impair decidualization, thereby jeopardizing implantation and early placental development. The etiology of RPL has been frequently linked to OS, which may function as a byproduct of microbial dysbiosis-driven chronic inflammatory activation [101,102]. Inflammatory signals from the microbiome may potentially affect the endometrium’s gene expression patterns and epigenetic regulation. Histone modification, DNA methylation, and transcriptional programs related to immunological tolerance and stromal cell differentiation can all be influenced by inflammatory cytokines and microbial metabolites. The repeated nature of pregnancy loss seen in RPL may have a mechanistic basis due to such epigenetic changes that may endure across cycles [41,67].

These molecular pathways act in concert to generate an endometrial environment marked by gene expression dysregulation, OS, and heightened inflammation. According to this theory, microbiome dysbiosis contributes to RPL by acting as an upstream cause of molecular and inflammatory disruptions that impair implantation and early placental development [15,103].

## 10. Methodological Challenges and Knowledge Gaps in Microbiome and Immune Research on RPL

Despite growing interest in the immunological and microbiome-related pathways involved in RPL, data interpretation and cross-study comparisons remain challenging because of several methodological limitations. These include variability in sampling techniques, analytical approaches, study design, and the integration of multi-layered biological data [12,104]. The low microbial biomass of endometrial samples is one of the main obstacles in research on the microbiome of the reproductive system. If strict controls are not used, low biomass increases susceptibility to reagent and environmental contamination, which can significantly affect sequencing results. Heterogeneity between research is further influenced by variations in bioinformatic pipelines, sequencing platforms, DNA extraction procedures, and sampling strategies [105,106].

The prevalence of cross-sectional study designs is another significant drawback. Most existing studies assess immunological features and microbiome composition at a single time point, limiting the ability to determine causal relationships among dysbiosis, immune dysregulation, and pregnancy loss. Although they are still rare, longitudinal studies that document dynamic changes during the menstrual cycle and the early stages of pregnancy are desperately needed. Another issue is the heterogeneity of patient populations [107].

Interpretation is complicated, and biologically significant relationships may be obscured by differences in the diagnostic criteria for RPL, the inclusion of sporadic vs. recurrent miscarriage cases, and the uneven exclusion of established etiologies (e.g., chromosomal, morphological, endocrine reasons) [2,6]. Differences in immune phenotyping methods further restrict comparison from an immunological standpoint. Despite mounting evidence that systemic immune profiles may not always represent local immunological conditions at the maternal–fetal interface, peripheral immune measures are commonly utilized as proxies for decidual immune status [108].

Crucially, very few studies combine microbiome, immunological, and genetic data from the same cohort. Understanding how genetic vulnerability, microbial signals, and immunological regulation interact to cause RPL is limited by the lack of multi-omic methods [20,41]. To improve mechanistic knowledge, further research utilizing standardized procedures, longitudinal sampling, and integrated genomic, immunologic, and microbiome analysis will be crucial. An overview of the genetic, immunological, microbiome-related, and molecular factors involved in RPL is presented in Table 2.

## 11. Clinical Implications and Translational Perspectives

There are significant clinical and translational ramifications to the growing research that links immune dysregulation, microbiome changes, and genetic predisposition to RPL. These findings highlight the limitations of traditional diagnostic approaches, which emphasize chromosomal, anatomical, and endocrine factors while leaving a substantial proportion of RPL cases unexplained [115,116]. It has been suggested that microbiome profiling of the endometrial and vaginal compartments could be used as an additional diagnostic method to identify women who are more likely to experience adverse reproductive outcomes [117].

However, the typical clinical use of microbiome-based diagnostics is currently limited by considerable diversity in collection techniques, analytical pipelines, and clinical goals. The incorporation of these approaches into clinical practice will require standardized methodologies and validation in carefully designed prospective cohorts [118,119].

Modulation of the microbiome–immune axis is an appealing but experimental approach from a therapeutic standpoint. There is still little solid data to support the effectiveness of interventions, including probiotics, antibiotics, dietary changes, and immune-targeted medicines in treating RPL. Crucially, indiscriminate modification of the immune system or microbiota may have unforeseen implications, highlighting the necessity for individualized and mechanistically informed strategies [116,120]. Patient classification may be made easier by the discovery of immunological and microbiome-related indicators, allowing for more focused research and treatment of unexplained RPL. These techniques could be used to differentiate between women whose immune–microbiome dysregulation is the main cause of pregnancy loss and those whose other causes are more prevalent [121].

In general, interdisciplinary cooperation, longitudinal study designs, and the integration of genetic, immunological, and microbiome data will be necessary to translate new mechanistic insights into clinical benefit. The field may advance from descriptive associations to precision-based treatments for repeated miscarriages as a result of these initiatives [122].

## 12. Conclusions

Genetic defects alone cannot adequately explain RPL, which is a complex and complicated reproductive conditions. While random pregnancy loss and a portion of recurrent instances are largely caused by embryonic aneuploidy and parental chromosomal abnormalities, there is growing evidence that immunological dysregulation and changes in the microbiome are important causes of unexplained RPL.

To influence endometrial receptivity, implantation success, and early placental development, genetic susceptibility interacts with immunological and microbiome-related variables. This review shows a multilayered pathophysiological framework. At the maternal–fetal interface, immune–microbiome crosstalk is identified as a key mechanism that connects environmental exposures to maternal immunological tolerance. Integrative methods that integrate genetic, immunological, and microbial investigations within well-characterized patient cohorts will be necessary to advance our understanding of RPL. To improve outcomes for women who experience RPL, such efforts are crucial for the creation of trustworthy biomarkers and focused therapy approaches.

## Figures and Tables

**Table 1:** Genetic contributions to sporadic versus recurrent pregnancy loss. This table shows the genetic factors linked to SPL and RPL. Embryonic aneuploidy is the main cause of SPL, while RPL is more often related to parental chromosomal rearrangements or euploid pregnancy loss, rather than fetal chromosomal abnormalities. The evidence strength is based on studies, cytogenetic analyses, and clinical guidelines.

Genetic Factor	SPL	RPL	Strength of Evidence
Embryonic aneuploidy [5]	Very common (≈50–70%)	Less frequent	Strong
Maternal age effect [24]	Strong association	Moderate association	Strong
Balanced chromosomal translocations [45]	Rare	Present in ~2–5% of couples	Strong
CNVs [46]	Occasional	Limited contribution	Moderate
Monogenic disorders [47]	Rare	Rare (selected cases)	Limited–Moderate
Euploid pregnancy loss [5]	Uncommon	Frequent	Moderate–Strong

**Table 2:** Integrated genetic and non-genetic contributors to RPL. This table summarizes genetic, epigenetic, immunological, microbiome-related, and molecular factors implicated in RPL. Genetic susceptibility represents an initiating layer but is insufficient to explain most cases of unexplained RPL, which arise from interactions among immune dysregulation, microbiome alterations, and downstream inflammatory and molecular pathways.

Biological Layer	Key Features in RPL	Impact on Pregnancy
Genetic susceptibility [27]	Lower prevalence of embryonic aneuploidy vs. sporadic loss; parental rearrangements in a subset	Genetics alone insufficient
Epigenetic regulation [109]	Altered decidual gene expression	Impaired decidualization
Immune dysregulation [57,110]	Increased NK cytotoxicity; Decreased Tregs; Th1/Th17 skewing	Loss of immune tolerance
Vaginal microbiome [15,20]	Reduced *Lactobacillus* dominance	Local inflammation
Endometrial microbiome [67,111]	Low-biomass microbial dysbiosis	Impaired implantation
Systemic microbiomes [112,113]	Gut and oral inflammatory signatures	Immune priming
Molecular pathways [3,114]	NF-κB activation; OS	Early placental failure

## Data Availability

No new data were created or analyzed in this study.

## Abbreviations

The following abbreviations are used in this manuscript:

RPL	Recurrent Pregnancy Loss
SPL	Sporadic Pregnancy Loss
PGT-A	Preimplantation Genetic Testing for Aneuploidy
dNK	Decidual Natural Killer
Tregs	Regulatory T cells
NF-κB	Nuclear Factor-κΒ
OS	Oxidative Stress
CNVs	Copy number Variants
